# Journal of Neuro Oncology: Diagnostic and therapeutic implications of IDH mutations in gliomas following the 2021 World Health Organization classification of CNS tumors

**DOI:** 10.1007/s11060-023-04317-3

**Published:** 2023-05-22

**Authors:** Tareq A. Juratli, Christine Jungk, Julie J. Miller

**Affiliations:** 1 Faculty of Medicine and University Hospital Carl Gustav Carus of Technische Universität Dresden, Department of Neurosurgery, Division of Neuro-Oncology, 01307 Dresden, Germany; 2 Translational Neuro-Oncology Laboratory, Department of Neurosurgery, Massachusetts General Hospital, Boston, MA USA; 3 Department of Neurosurgery, University Hospital Heidelberg, 69120 Heidelberg, Germany; 4 Stephen E. and Catherine Pappas Center for Neuro-Oncology, Department of Neurology, Massachusetts General Hospital, Boston, MA USA

## Guest Editorial

The discovery of mutations in the IDH1 and IDH2 genes in gliomas has significantly impacted the classification and treatment of these tumors [1, 2]. While histological grading has traditionally been used to predict prognosis in gliomas, it is now evident that IDH mutation status provides a more accurate indicator of a patient’s clinical course. In recognition of this, the WHO 2016 classification of CNS tumors has defined IDH-mutant gliomas as a distinct entity, marking a paradigm shift in tumor categorization [3]. However, despite the significant impact of IDH mutation status on prognosis, the post-treatment clinical course of IDH-mutant gliomas remains highly variable [4, 5]. To gain a better understanding of the biological and clinical factors contributing to this variability, researchers have identified other recurrent molecular abnormalities and altered intracellular signaling pathways in IDH-mutant gliomas [6, 7]. The 2021 WHO classification has incorporated some of these molecular markers to better characterize IDH-mutant astrocytoma and oligodendroglioma and predict treatment response [8]. The integration of molecular and clinical data has paved the way for the development of new diagnostic tools and therapeutic strategies targeting oncogenic signaling pathways for the treatment of IDH-mutant patients.

This special issue features cutting-edge research in the field of IDH-mutant gliomas, including original research articles and expert reviews covering various aspects of basic, translational, and clinical research. The focus is on genomic discoveries and recent diagnostic techniques in gliomas, with several articles shedding light on novel biomarkers, therapeutic advancements, and neuropsychological aspects.

In his article titled “Updates on the WHO diagnosis of IDH-mutant glioma,“ David Reuss provides a summary of the changes related to IDH-mutant gliomas according to the updated WHO classification [9]. He discusses unresolved issues and future perspectives, highlighting the clinical relevance of molecular features that are now used for both classification and grading. Reuss concludes that further advancements in our understanding of the molecular landscape of IDH-mutant gliomas are expected to refine classification and grading in the future.

Kessler and colleagues, in their review titled “Conventional and emerging treatments of astrocytomas and oligodendrogliomas,“ discuss treatment approaches for astrocytomas and oligodendrogliomas, including maximal safe resection, combined radiochemotherapy, and surgery alone based on histopathological diagnosis and prognostic factors [10]. However, they note that there is a lack of effective treatments for progressive disease, and ongoing investigations into immune- and targeted therapies aim to optimize treatment outcomes. They also highlight that IDH mutation is a target for small molecule inhibition and immune therapy in diffuse astrocytomas and oligodendrogliomas, while the BRAF pathway is targetable in circumscribed gliomas [10].

Notably, data on the treatment of IDH-mutant patients older than 60 years with lower-grade gliomas are limited. The study by Dao Trong and colleagues challenges the notion that elderly patients with lower-grade glioma (LGG) have poor outcomes by analyzing a contemporary cohort of patients with specific molecular characteristics [11]. They found that elderly patients had comparable progression-free survival (PFS) to younger patients, and favorable surgical and survival outcomes were achieved in the elderly patients, supporting the recommendation of intensified treatment, including maximal safe resection, in elderly patients whenever feasible [11].

When it comes to targeted diagnostics in IDH-mutant gliomas, PET imaging with radiolabeled amino acids, along with MRI, is a valuable diagnostic tool for managing brain tumors. Many of these IDH-mutant gliomas do not show contrast enhancement on MRI, making diagnosis and treatment planning challenging. The review “Clinical applications and prospects of PET imaging in patients with IDH-mutant gliomas” by Michael Wollring and colleagues discusses PET studies in glioma patients with an isocitrate dehydrogenase (IDH) gene mutation [12]. The review focuses on the role of amino acid PET in this context, as well as the potential use of PET probes targeting the IDH mutation for response assessment in clinical trials using IDH inhibitors for treating IDH-mutant gliomas [12].

From a therapeutic perspective, Proton beam radiotherapy (PRT) is another therapeutic option for IDH-mutant gliomas. Eichkorn and colleagues, in their study titled “Analysis of safety and efficacy of proton radiotherapy for IDH-mutated glioma WHO grade 2 and 3,“ analyzed 194 patients treated with PRT from 2010 to 2020 [13]. They found that PRT was effective in treating IDH-mutated glioma WHO grade 2 and 3, but the risk of radiation-induced contrast enhancements (RICE) differed with tumor grading and was higher in older patients [13]. Similar findings were reported by Xianxin Qiu et al., who found that Proton radiotherapy resulted in favorable outcomes with acceptable adverse effects in patients with IDH-mutant diffuse gliomas [14].

Another promising avenue of research is the identification of biomarkers that can predict treatment response in IDH-mutant gliomas. By gaining a better understanding of the molecular mechanisms underlying treatment resistance, researchers aim to identify new targets for therapy and improve patient outcomes. In their article titled “DNA damage in IDH-mutant gliomas: mechanisms and clinical implications,“ Shi and colleagues describe the current understanding of how IDH mutations impact DNA damage in glioma [15]. The effects of mutant IDH on DNA damage can be categorized based on gene expression changes, sensitivity to alkylating agents, disruption of homologous repair and response to oxidative stress [15].

Finally, the neuropsychological impact of IDH-mutant gliomas is an area of growing interest. These tumors can affect cognitive function, mood, and quality of life, and it is important to better understand these effects in order to provide appropriate support and care for patients and their families. In their article entitled “Cognitive issues in patients with IDH mutant gliomas: from neuroscience to clinical neuropsychology“, Michael Parsons and David Sabeseitz summarize the current understanding of cognitive symptoms in patients with IDH-Mutant gliomas [16]. The article reviews relevant literature on the influence of IDH-mutant tumors and their treatment on cognition, and provides guidance on managing these symptoms in patients [16]. The results suggest that patients with IDH-mutant gliomas have a favorable cognitive profile compared to those with IDH-wild type tumors, possibly due to the slower growth rate of IDH-mutant tumors. The article also highlights the importance of integrating intra-operative mapping during surgery to mitigate cognitive decline and instituting neuropsychological assessment as part of long-term care for patients with IDH-Mutant gliomas [16]. Given the recent classification of gliomas based on IDH mutation and the long time course of this disease, the article emphasizes the need for a thoughtful and comprehensive strategy to study patient outcomes and devise methods of cognitive risk reduction.

In conclusion, the discovery of mutations in the IDH1 and IDH2 genes has revolutionized our understanding of lower-grade gliomas and opened up new avenues for diagnosis and treatment. A crucial area of research is the development of targeted therapies that exploit the specific vulnerabilities of IDH-mutant tumors. This special issue of the journal provides a comprehensive overview of the latest research in this field and highlights the exciting potential for continued progress in the years to come.
